# Long-Term Survival Among Histological Subtypes in Advanced Epithelial Ovarian Cancer: Population-Based Study Using the Surveillance, Epidemiology, and End Results Database

**DOI:** 10.2196/25976

**Published:** 2021-11-17

**Authors:** Shi-Ping Yang, Hui-Luan Su, Xiu-Bei Chen, Li Hua, Jian-Xian Chen, Min Hu, Jian Lei, San-Gang Wu, Juan Zhou

**Affiliations:** 1 Department of Radiation Oncology Hainan General Hospital (Hainan Affiliated Hospital of Hainan Medical University) Haikou China; 2 Department of Nephrology Hainan General Hospital (Hainan Affiliated Hospital of Hainan Medical University) Haikou China; 3 Department of Radiation Oncology The First Affiliated Hospital of Xiamen University Xiamen China; 4 Department of Obstetrics and Gynecology The First Affiliated Hospital of Xiamen University Xiamen China; 5 Department of Medical Oncology People’s Hospital of Baise Baise China

**Keywords:** ovarian epithelial carcinoma, survivors, histology, survival rate, survival, ovarian, cancer, surveillance, epidemiology, women’s health, reproductive health, Surveillance, Epidemiology, and End Results, ovary, oncology, survivorship, long-term outcome, epithelial

## Abstract

**Background:**

Actual long-term survival rates for advanced epithelial ovarian cancer (EOC) are rarely reported.

**Objective:**

This study aimed to assess the role of histological subtypes in predicting the prognosis among long-term survivors (≥5 years) of advanced EOC.

**Methods:**

We performed a retrospective analysis of data among patients with stage III-IV EOC diagnosed from 2000 to 2014 using the Surveillance, Epidemiology, and End Results cancer data of the United States. We used the chi-square test, Kaplan–Meier analysis, and multivariate Cox proportional hazards model for the analyses.

**Results:**

We included 8050 patients in this study, including 6929 (86.1%), 743 (9.2%), 237 (2.9%), and 141 (1.8%) patients with serous, endometrioid, clear cell, and mucinous tumors, respectively. With a median follow-up of 91 months, the most common cause of death was primary ovarian cancer (80.3%), followed by other cancers (8.1%), other causes of death (7.3%), cardiac-related death (3.2%), and nonmalignant pulmonary disease (3.2%). Patients with the serous subtype were more likely to die from primary ovarian cancer, and patients with the mucinous subtype were more likely to die from other cancers and cardiac-related disease. Multivariate Cox analysis showed that patients with endometrioid (hazard ratio [HR] 0.534, *P*<.001), mucinous (HR 0.454, *P*<.001), and clear cell (HR 0.563, *P*<.001) subtypes showed better ovarian cancer-specific survival than those with the serous subtype. Similar results were found regarding overall survival. However, ovarian cancer–specific survival and overall survival were comparable among those with endometrioid, clear cell, and mucinous tumors.

**Conclusions:**

Ovarian cancer remains the primary cause of death in long-term ovarian cancer survivors. Moreover, the probability of death was significantly different among those with different histological subtypes. It is important for clinicians to individualize the surveillance program for long-term ovarian cancer survivors.

## Introduction

### Background

Advanced stage (stage III-IV) epithelial ovarian cancer (EOC) is usually incurable. However, approximately 25% and 15% of patients with EOC survive for >5 years and >10 years, respectively [1-4]. Although it largely remains unknown why long-term survivors have a better outcome, investigating the underlying mechanisms or factors is key for developing individualized follow-up strategies for patients with EOC. Several epidemiological, clinical, and genetic factors have been associated with the long-term survival of patients with EOC [5,6].

Based on the World Health Organization classification of tumors of female reproductive organs, which was published in 2014, EOC can be classified into five histological subtypes: high-grade serous, low-grade serous, endometrioid, clear cell, and mucinous [7]. A previous study using the California Cancer Registry reported that the nonserous subtype is an independent predictor of long-term survival in EOC; favorable prognoses were observed among patients with the endometrioid, mucinous, and clear cell subtypes than in those with the serous subtype [3]. However, the same study also included patients with early-stage EOC, and it may thus not reﬂect the true long-term survival characteristics of patients with advanced-stage EOC.

The endometrioid and mucinous subtypes are typically low-grade and early-stage, and patients with these subtypes show a better outcome than those with the high-grade serous subtype [8-10]. Moreover, although the clear cell subtype exhibits high-grade features, it is more likely to present with early-stage disease and is associated with a better outcome than high-grade serous cancers [11]. However, several studies, including ours, have confirmed that advanced mucinous and clear cell cancers display aggressive behavior, and patients with these have lower survival than those with high-grade serous tumors, which can perhaps be attributed to chemoresistance characteristics [12-18]. Accordingly, this study aimed to assess the role of histological subtypes in predicting the prognosis of long-term survivors (≥5 years) of advanced EOC.

## Methods

**Figure 1:**
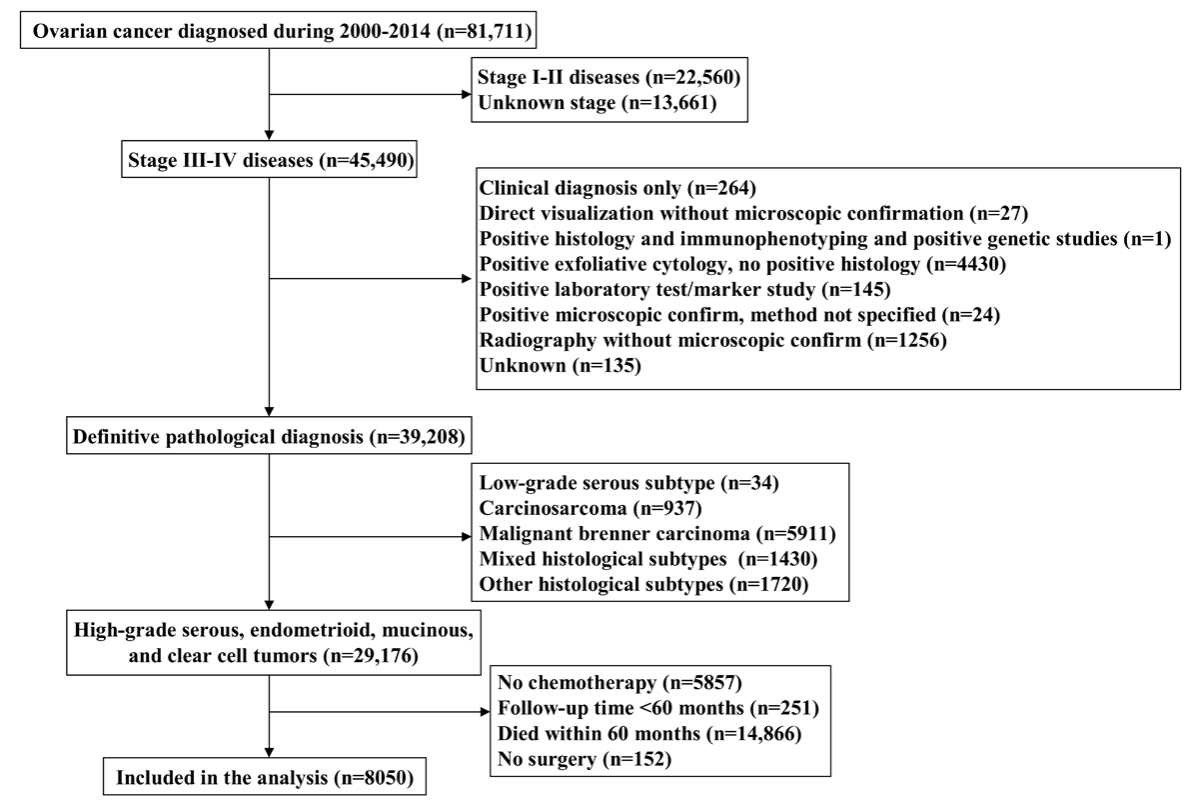
Flow diagram of the study cohort.

We extracted EOC data from the Surveillance, Epidemiology, and End Results (SEER) database of the United States, which is a publicly available database and contains deidentified information on cancer incidence, demographic and clinicopathological variables, patterns of the first course of treatment, and survival data [19]. We selected patients of all ages who were diagnosed with stage III-IV EOC from 2000 to 2014. We included long-term ovarian cancer survivors (≥5 years) in this study. The patient selection flowchart is shown in Figure 1. We included those with high-grade serous, endometrioid, clear cell, and mucinous subtypes. Patients who did not undergo any surgery or did not receive chemotherapy were excluded. In addition, patients who died within 60 months after the diagnosis of ovarian cancer or who had follow-up times of <60 months were also excluded. The analysis of the SEER database was exempt from the approval process of the institutional review board considering the presence of deidentified patient information.

A total of 29,176 patients with stage III-IV EOC were identified. Of these patients, 5857 did not receive chemotherapy, 251 patients had a follow-up time of <60 months, 14,866 patients died within 60 months, and 152 patients did not receive surgery. A total of 8050 EOC patients with ≥5 years’ survival were included in this study.

This study could be used to assist physicians in prognostic assessment at the time of diagnosis of EOC and help physicians better understand EOC from the long-term survivors to prolong the survival time of short-term survivors. The SEER program collects long-term follow-up cancer data, thus allowing us to assess the long-term survivors of EOC. We included the following demographic, clinicopathological, and treatment variables: age at diagnosis, race, stage, histological subtype, and nodal status. The definition of the staging system was based on the American Joint Committee on Cancer (AJCC) sixth edition staging system. The primary endpoints of this study were ovarian cancer–specific survival (OCSS) and overall survival (OS). OCSS was defined as the time from diagnosis to death due to ovarian cancer, censoring at the date of last contact, or nonovarian cancer related–death. OS was defined as the time from diagnosis of ovarian cancer to the death from any cause.

The association among demographic, clinicopathological, and treatment variables for the histological subtypes was compared using the chi-square test and the Fisher exact test. Survival comparisons were made using Kaplan–Meier analysis and compared using the log-rank test. Multivariate Cox proportional hazards model was used to determine the prognostic factors associated with OCSS and OS. Prognostic factors with statistical significance on univariate analyses were entered into multivariate analyses. The proportional hazard assumption was tested both graphically and using the Schoenfield residual test to address whether our data met the proportional odds assumption, allowing for the use of the Cox proportional hazards model. Sensitivity analyses were performed on the basis of the age at diagnosis, race, AJCC staging, and nodal status to investigate the effect of the histological subtype on survival outcomes. SPSS (version 22.0, IBM Corp) and Stata/SE (version 14, StataCorp) were used for analyses, and *P*<.05 indicated statistical significance.

## Results

### Patient Characteristics

Patient characteristics and causes of death data are listed in Table 1. Of the entire cohort, 6929 (86.1%), 743 (9.2%), 237 (2.9%), and 141 (1.8%) showed the presence of serous, endometrioid, clear cell, and mucinous tumors, respectively. The majority of patients were aged ≥50 years (76.8%, n=6138), white race (86.6%, n=6975), stage III disease (75.6%, n=6164), and nodal negative disease (57.8%, n=4651). Patients with the serous subtype were more likely to be older (*P*<.001) and diagnosed with stage IV disease (*P*<.001) than those with the other 3 histological subtypes. In addition, patients with the serous subtype had a higher risk of regional lymph node metastasis than those with the endometrioid and mucinous subtypes (35.7% vs 26.2%-31.5%), while those with the clear cell subtype had a higher risk of regional lymph node metastasis than those with the other 3 histological subtypes (42.6% vs 26.2%-35.7%) (*P*<.001).

**Table 1:** Baseline patient characteristics and causes of death by histological subtype in patients with epithelial ovarian cancer diagnosed from 2000 to 2014 using the Surveillance, Epidemiology, and End Results cancer data of the United States.

Variables		Patients, n	Serous subtype, n (%)	Endometrioid subtype, n (%)	Mucinous subtype, n (%)	Clear cell subtype, n (%)	*P* value
**Age (years)**							<.001
	<50	1867	1528 (22.1)	231 (31.1)	51 (36.2)	57 (24.1)	
	50-64	3662	3146 (45.4)	323 (43.5)	64 (45.4)	129 (54.4)	
	≥65	2521	2255 (32.5)	189 (25.4)	26 (18.4)	51 (21.5)	
**Race**							<.001
	White	6975	6037 (87.1)	616 (82.9)	124 (87.9)	198 (83.5)	
	Black	420	371 (5.4)	41 (5.5)	3 (2.1)	5 (2.1)	
	Other	655	521 (7.5)	86 (11.6)	14 (9.9)	34 (14.3)	
**American Joint Committee on Cancer stage**							<.001
	III	6164	5235 (75.6)	629 (84.7)	113 (80.1)	187 (78.9)	
	IV	1886	1694 (24.4)	114 (15.3)	28 (19.9)	50 (21.1)	
**Nodal status**							<.001
	Negative	4651	3954 (57.1)	477 (64.2)	99 (70.2)	121 (51.1)	
	Positive	2849	3477 (35.7)	234 (31.5)	37 (26.2)	101 (42.6)	
	Unknown	550	498 (7.2)	32 (4.3)	5 (3.5)	15 (6.3)	
**Death (n=3874)**							<.001
	Primary ovarian cancer	3111	2819 (81.8)	199 (68.4)	33 (61.1)	60 (73.2)	
	Other cancers	312	266 (7.7)	37 (12.7)	7 (13.0)	2 (2.4)	
	Cardiac death	123	96 (2.8)	12 (4.1)	9 (16.7)	6 (7.3)	
	Pulmonary deaths	44	36 (1.0)	2 (0.7)	1 (1.9)	5 (6.1)	
	Other causes	284	230 (6.7)	41 (14.1)	4 (7.4)	9 (11.0)	

### Causes of Death in Long-Term Ovarian Cancer Survivors

This cohort included 5967 patients surviving for ≥5 years and <10 years (60-119 months) and 2353 patients surviving ≥10 years (≥120 months). With a median follow-up of 91 (range 60-227) months, a total of 3874 deaths were recorded. The most common cause of death was primary ovarian cancer (80.3%, n=3111), followed by other cancers (8.1%, n=312), other causes of death (7.3%, n=284), cardiac-related death (3.2%, n=123), and nonmalignant pulmonary disease (3.2%). Patients with the serous subtype were more likely to die from primary ovarian cancer, and those with the mucinous subtype were more likely to die from other cancers and cardiac-related disease (Table 1). Among those surviving ≥5 years and <10 years, 83.3% died owing to primary ovarian cancer, 7.4% died owing to other cancers, 6.0% died owing to other causes, 2.4% died owing to cardiac disease, and 0.9% died owing to nonmalignant pulmonary disease. For patients surviving ≥10 years, 63.0% died owing to primary ovarian cancer, 14.9% died owing to other causes, 12.1% died owing to other cancers, 7.7% died owing to cardiac disease, and 2.3% died owing to nonmalignant pulmonary disease.

**Figure 2:**
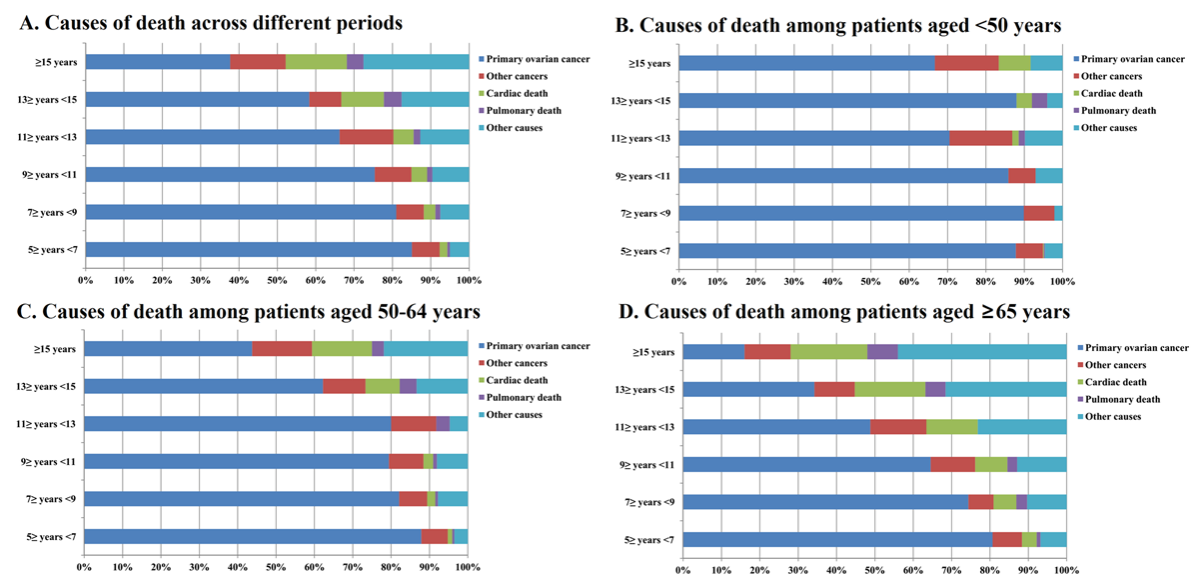
Causes of death after stratification by years of survival after diagnosis of epithelial ovarian cancer for long-term survivors: (A) the entire cohort; (B) patients aged <50 years; (C) patients aged 50-64 years; and (D) patients aged ≥65 years.

The causes of death after stratification by years of survival after diagnosis of EOC for long-term survivors are detailed in Figure 2. Figure 2A shows that in the entire cohort, with an increase in the time from diagnosis, death because of ovarian cancer–related causes decreases, while death owing to cardiac disease and other causes increases. In patients aged <50 years and 50-64 years, death due to ovarian cancer–related disease remained the main cause of death with an increase in the time from diagnosis, and death from primary ovarian cancer was still significant among other causes of death even 15 years after diagnosis of ovarian cancer (Figure 2B and 2C). Among those aged ≥65 years, death because of ovarian cancer–related causes decreased and death due to cardiac disease and other causes increased (Figure 2D).

### Survival Outcomes and Prognostic Analyses

**Figure 3:**
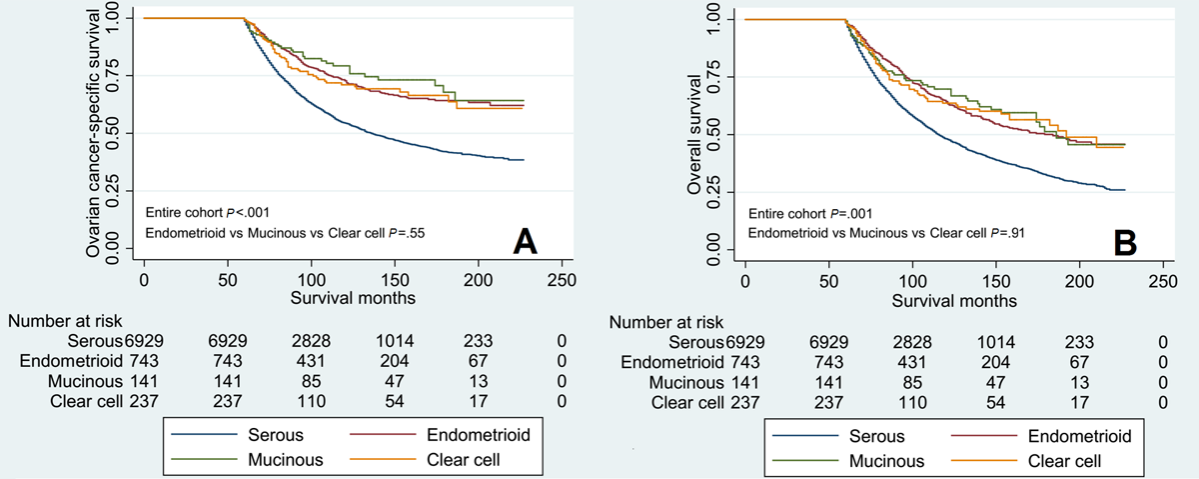
Comparison of ovarian cancer–specific survival (A) and overall survival (B) among the 4 histological subtypes of the epithelial ovarian cancer diagnosed from 2000 to 2014 using the Surveillance, Epidemiology, and End Results cancer data of the United States.

Kaplan–Meier analysis was conducted to compare the survival curves among the 4 histological subtypes (Figure 3). The results showed that serous subtype had a significantly lower OCSS (*P*<.001) and OS (*P*<.001) compared to those with the other 3 histological subtypes, while comparable OCSS (*P*=.55) (Figure 3A) and OS (*P*=.91) (Figure 3B) were observed among those with endometrioid, mucinous, and clear cell cancers.

**Figure 4:**
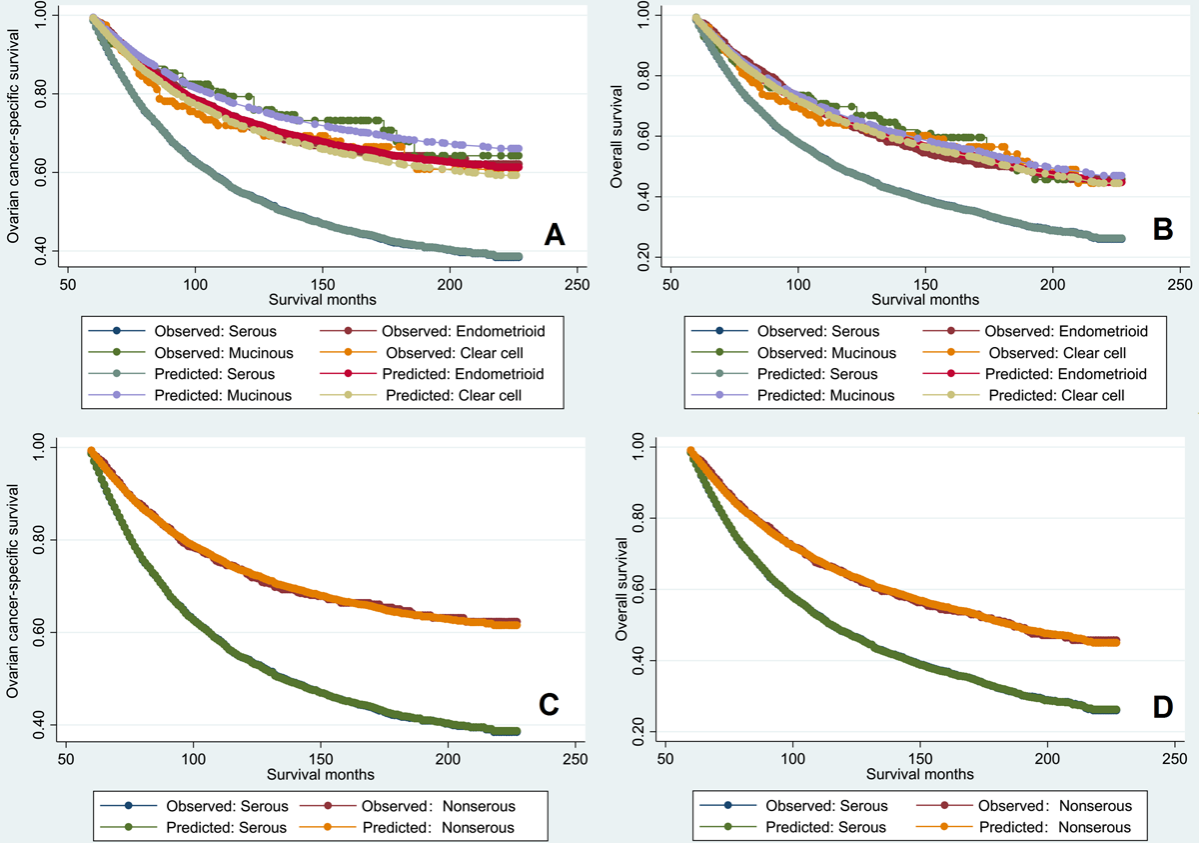
The evaluation of the proportional hazards assumption in ovarian cancer-specific survival (A and C) and overall survival (B and D) among the different histological subtypes of the epithelial ovarian cancer from 2000 to 2014 using the Surveillance, Epidemiology, and End Results cancer data of the United States.

Univariate and multivariate analyses were used to determine the prognostic factors related to OCSS and OS. Univariate analyses showed that age at diagnosis, AJCC staging, nodal status, and histological subtype were the prognostic factors associated with OCSS and OS (Tables 2 and 3). The results showed that age at diagnosis, AJCC staging, nodal status, and histological subtype were also the independent prognostic factors associated with OCSS and OS. Patients with endometrioid (hazard ratio [HR] 0.534, 95% CI 0.462-0.617, *P*<.001), mucinous (HR 0.454, 95% CI 0.322-0.641, *P*<.001), and clear cell (HR 0.563, 95% CI 0.436-0.727, *P*<.001) subtypes showed better OCSS than those with the serous subtype. Similar results were obtained regarding OS. Using clear cell tumor as a reference, similar OCSS and OS were observed in endometrioid (OCSS: HR 0.949, 95% CI 0.711-1.267, *P*=.72; OS: HR 1.014, 95% CI 0.793-1.295, *P*=.91) and mucinous cancers (OCSS: HR 0.807, 95% CI 0.528-1.235, *P*=.32; OS: HR 0.969, 95% CI 0.687-1.366, *P*=.86) compared to those with clear cell tumor. The effect of the histological subtype on OCSS (Figure 4A) and OS (Figure 4B) met the proportional hazard assumption, which showed that the constant HRs from the Cox model were reliable.

**Figure 5:**
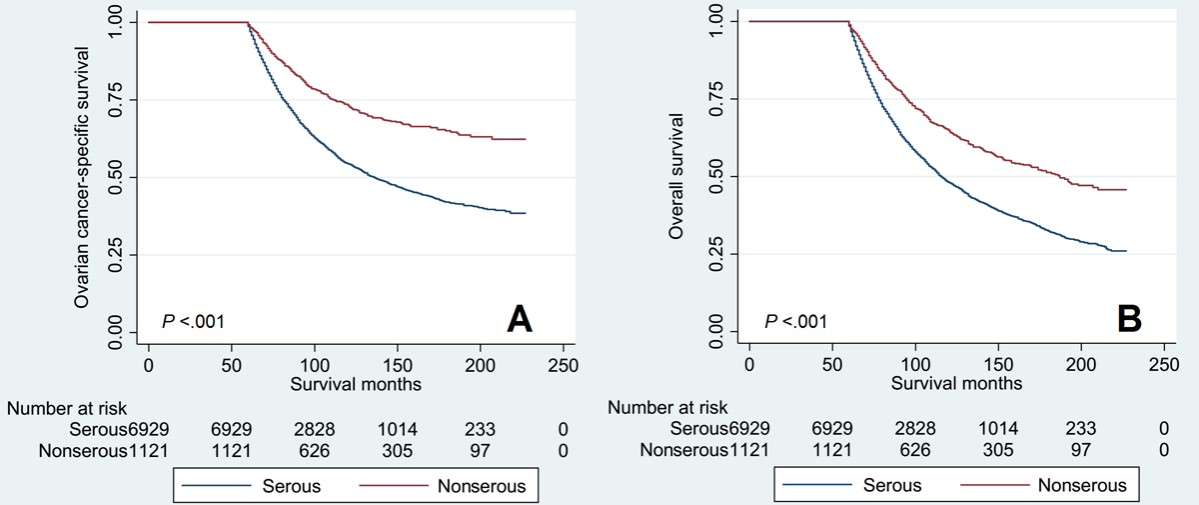
Ovarian cancer-specific survival curves (A) and overall survival curves (B) between serous cancer and non-serous cancer from 2000 to 2014 using the Surveillance, Epidemiology, and End Results cancer data of the United States.

Since we observed similar survival outcomes among patients with endometrioid, mucinous, and clear cell cancers, we combined these histological subtypes under the nonserous subtype to compare the survival outcomes with those of serous cancers. Kaplan–Meier analysis showed that patients with the serous subtype had a significantly lower OCSS (*P*<.001) (Figure 5A) and OS (*P*<.001) (Figure 5B) than those with nonserous tumors.

Sensitivity analyses were focused on age at diagnosis, race, AJCC staging, and nodal status to investigate the effect of histology on survival outcomes (Table 4). The obtained results indicated that patients with the serous subtype had lower OCSS and OS than those with the nonserous subtype, stratified by age at diagnosis, stage at diagnosis, and nodal stage. Among White patients and those of other races, the serous subtype was characterized with lower OCSS and OS than the nonserous subtype. Between the serous and nonserous subtypes, the OCSS and OS were comparable among Black patients. The effect of the histology on OCSS (Figure 4C) and OS (Figure 4D) met the proportional hazard assumption, which indicated that the constant HRs ratios from the Cox model were reliable.

**Table 2:** Univariate and multivariate survival analyses of ovarian cancer–specific survival in long-term survivors of the epithelial ovarian cancer diagnosed from 2000 to 2014 using the Surveillance, Epidemiology, and End Results cancer data of the United States.

Variables		Univariate survival analysis		Multivariate survival analysis	
		Hazard ratio (95% CI)	*P* value	Hazard ratio (95% CI)	*P* value
**Age (years)**					
	<50	1 (reference)	Reference	1 (reference)	Reference
	50-64	1.137 (1.037-1.246)	.006	1.091 (0.995-1.196)	.06
	≥65	1.457 (1.323-1.606)	<.001	1.361 (1.234-1.500)	<.001
**Race**					
	White	1 (reference)	Reference	—^a^	—
	Black	0.949 (0.804-1.119)	.53	—	—
	Other	0.913 (0.797-1.044)	.18	—	—
**American Joint Committee on Cancer stage**					
	III	1 (reference)	Reference	1 (reference)	Reference
	IV	1.355 (1.252-1.467)	<.001	1.224 (1.125-1.333)	<.001
**Nodal status**					
	Negative	1 (reference)	Reference	1 (reference)	Reference
	Positive	0.847 (0.784-0.916)	<.001	0.844 (0.781-0.912)	<.001
	Unknown	1.511 (1.339-1.705)	<.001	1.309 (1.149-1.490)	<.001
**Histological subtypes**					
	Serous	1 (reference)	Reference	1 (reference)	Reference
	Endometrioid	0.515 (0.446-0.595)	<.001	0.534 (0.462-0.617)	<.001
	Mucinous	0.436 (0.309-0.615)	<.001	0.454 (0.322-0.641)	<.001
	Clear cell	0.550 (0.426-0.711)	<.001	0.563 (0.436-0.727)	<.001

^a^—: not determined.

**Table 3:** Univariate and multivariate survival analyses of overall survival among long-term survivors of the epithelial ovarian cancer diagnosed from 2000 to 2014 using the Surveillance, Epidemiology, and End Results cancer data of the United States.

Variables		Univariate survival analysis		Multivariate survival analysis	
		Hazard ratio (95% CI)	*P* value	Hazard ratio (95% CI)	*P* value
**Age (years)**					
	<50	1 (reference)	Reference	1 (reference)	Reference
	50-64	1.188 (1.091-1.293)	<.001	1.149 (1.055-1.251)	.001
	≥65	1.763 (1.615-1.924)	<.001	1.668 (1.527-1.821)	<.001
**Race**					
	White	1 (reference)	Reference	—^a^	—
	Black	0.970 (0.838-1.123)	.69	—	—
	Other	0.916 (0.812-1.034)	.16	—	—
**American Joint Committee on Cancer stage**					
	III	1 (reference)	Reference	1 (reference)	Reference
	IV	1.349 (1.257-1.448)	<.001	1.222 (1.132-1.319)	<.001
**Nodal status**					
	Negative	1 (reference)	Reference	1 (reference)	Reference
	Positive	0.853 (0.796-0.915)	<.001	0.863 (0.805-0.925)	<.001
	Unknown	1.484 (1.330-1.655)	<.001	1.298 (1.154-1.459)	<.001
**Histological subtypes**					
	Serous	1 (reference)	Reference	1 (reference)	Reference
	Endometrioid	0.601 (0.533-0.677)	<.001	0.632 (0.561-0.713)	<.001
	Mucinous	0.566 (0.432-0.740)	<.001	0.604 (0.461-0.791)	<.001
	Clear cell	0.606 (0.487-0.754)	<.001	0.624 (0.501-0.776)	<.001

^a^—: not determined.

**Table 4:** Stratified analyses of the multivariable-adjusted hazard ratios and 95% CIs for ovarian cancer–specific survival and overall survival by histological subtype (serous vs nonserous) among long-term survivors of the epithelial ovarian cancer diagnosed from 2000 to 2014 using the Surveillance, Epidemiology, and End Results cancer data of the United States.

Variables (serous vs nonserous)	Ovarian cancer–specific survival		Overall survival	
	Hazard ratio (95% CI)	*P* value	Hazard ratio (95% CI)	*P* value
Aged <50 years	2.381 (1.861-3.046)	<.001	2.088 (1.679-2.596)	<.001
Aged 50-64 years	1.809 (1.514-2.161)	<.001	1.516 (1.304-1.763)	<.001
Aged ≥65 years	1.636 (1.311-2.041)	<.001	1.414 (1.188-1.683)	<.001
White patients	1.797 (1.580-2.044)	<.001	1.569 (1.407-1.749)	<.001
Black patients	1.383 (0.799-2.395)	.25	1.088 (0.692-1.712)	.72
Patients of other race	3.672 (2.294-5.879)	<.001	2.276 (1.609-3.219)	<.001
American Joint Committee on Cancer stage III disease	1.715 (1.503-1.958)	<.001	1.507 (1.347-1.684)	<.001
American Joint Committee on Cancer stage IV disease	2.861 (2.102-3.894)	<.001	1.990 (1.575-2.515)	<.001
Nodal negative disease	1.832 (1.578-2.128)	<.001	1.593 (1.403-1.809)	<.001
Nodal positive disease	1.850 (1.475-2.319)	<.001	1.522 (1.264-1.833)	<.001

## Discussion

### Principal Findings

Herein we used the SEER database to assess the role of histological subtypes in predicting the survival outcome among long-term survivors (≥5 years) of advanced EOC. Our results indicate that ovarian cancer remains the primary cause of death among long-term ovarian cancer survivors. Moreover, patients with endometrioid, clear cell, and mucinous tumors showed a significant improvement in OCSS and OS compared to those with serous tumors. This study provides a unique opportunity to determine the characteristics of long-term survivors of advanced EOC.

There exist limited studies that have explored factors associated with long-term survival in EOC [6]. Such studies have reported that long-term survival is associated with various factors such as younger age at diagnosis, earlier clinicopathologic stage, absence of ascites, lower grade, earlier stage, nonserous histology, and lower CA125 levels [3-5,19,20]. In our study, we used a population-based cohort, and our results indicate that younger age, stage III disease, and nonserous cancers were associated with long-term survival in EOC. Our results add to the current knowledge on the prognostic factors for the long-term survivors of EOC. In addition, epidemiological factors such as low body mass index, not smoking, parity, and individual exhauster-scored conditions have been associated with long-term survival in EOC [5,21]. However, owing to the limitation of the SEER database, we could not identify these epidemiological factors.

In our previous studies, we have found a markedly increased mortality rate among patients with stage III-IV mucinous and clear cell cancers, but better survival among those with serous and endometrioid cancers [13,22]. These results concur with those reported by previous studies on stage III-IV EOC [13,15,18,20,23,24]. The aggressive behavior and impaired response to taxanes and platinum-based chemotherapy in the case of mucinous and clear cell carcinomas may be the core reason for these findings [16,25,26]. However, a study using the California Cancer Registry and including patients diagnosed with EOC between 1994 and 2001 reported that nonserous subtypes, including endometrioid, clear cell, and mucinous carcinomas, were significant predictors of long-term survival [3]; this study included patients with early-stage EOC, and it may hence not reﬂect the true long-term survival characteristics of patients with advanced-stage EOC.

The prognostic role of histological subtypes on the survival outcome among long-term survivors of advanced EOC has been explored by limited studies. A previous study by Son et al [27] reported that 91% of deaths occurred within 8 years, and that survival for 8 years may represent the prognostic inﬂection point for long-term survival in advanced EOC. However, only 11 patients survived for >8 years in the Son et al’s [27] study. In our study, approximately 80% of ovarian cancer–related deaths occurred in <5 years. Among long-term survivors (who survived for ≥5 years) (n=8050), 3874 patients died of any causes during follow-up, and the majority of patients died from ovarian cancer–related disease, particularly those with the serous subtype. Our results suggest that although the peak of ovarian cancer–related deaths occurred within 5 years, intensive follow-up is required for long-term survivors.

Several studies, including ours, have indicated that the survival outcome of clear cell and mucinous cancers were significantly inferior to that of serous cancers in advanced-stage disease [12-16]. However, in this study, the OCSS and OS for clear cell and mucinous cancers were significantly longer than those for serous cancers in long-term survivors, suggesting that the prognostic effect of the histological subtype on EOC survival changed upon extensive follow-up. Therefore, surveillance options tailored depending on the nature of the histological subtype of EOC should be considered in future studies. The mechanisms underlying this more aggressive course in early, but not in long-term, outcomes for mucinous and clear cell cancers in advanced-stage disease have not been studied in detail. Failure to respond to chemotherapy could contribute to poorer survival in clear cell and mucinous cancers with respect to early outcome [28], while the risk of death in patients with clear cell and mucinous cancers may be significantly reduced upon extensive follow-up [3]. Genetic signatures could further our understanding of the potential biological differences between short- and long-term survivors. However, the current evidence lacks consistency, limiting the reproducibility and clinical use of molecular markers [6].

### Strengths and Limitations

There were several limitations to our study. First, this is a retrospective study; hence, we could not exclude all potential selection biases. Second, information on chemotherapy regimens, administered dose, number of chemotherapy cycles, and completeness of chemotherapy were unavailable in the SEER database. Third, the size of residual tumors (before 2010), patterns of disease recurrence, and strategy of treatment after disease progression were also not recorded in the SEER database. Moreover, this database lacks a central review for histological subtype. On the other hand, the strengths of this study include its population-based design. This study involved a relatively large cohort of patients with EOC, with the data representing a real-world scenario. Furthermore, our results are expected to expand the current knowledge on the biological behavior of EOC by various histological subtypes after extensive follow-up. Further studies focusing on the prognostic factors regarding long-term survivors of EOC are needed.

### Conclusions

In conclusion, our study suggests that ovarian cancer remains the primary cause of death among long-term ovarian cancer survivors. Moreover, the probability of death is significantly different among those with different histological subtypes of EOC. It is important for clinicians to individualize the surveillance program for long-term ovarian cancer survivors. Further studies using diverse cohorts are warranted to confirm our findings and expand our understanding.

## Abbreviations

AJCC: American Joint Committee on Cancer
EOC: epithelial ovarian cancer
HR: hazard ratio
OCSS: ovarian cancer–specific survival
OS: overall survival
SEER: Surveillance, Epidemiology, and End Results
